# A systems science approach to identifying data gaps in national data sources on adolescent suicidal ideation and suicide attempt in the United States

**DOI:** 10.1186/s12889-023-15320-8

**Published:** 2023-04-01

**Authors:** Philippe J. Giabbanelli, Ketra L. Rice, Nisha Nataraj, Margaret M. Brown, Christopher R. Harper

**Affiliations:** 1 Department of Computer Science and Software Engineering, Miami University, 205W Benton Hall, High St, Oxford, OH 45056 USA; 2 National Center for Injury Prevention and Control, Centers for Disease Control and Prevention (CDC), Atlanta, GA USA; 3 Defense Suicide Prevention Office (DSPO), Department of Defense, Washington, DC USA

**Keywords:** Causal map, Data collection, Surveillance system, Youth suicide

## Abstract

**Background:**

Suicide is currently the second leading cause of death among adolescents ages 10–14, and third leading cause of death among adolescents ages 15–19 in the United States (U.S). Although we have numerous U.S. based surveillance systems and survey data sources, the coverage offered by these data with regard to the complexity of youth suicide had yet to be examined. The recent release of a comprehensive systems map for adolescent suicide provides an opportunity to contrast the content of surveillance systems and surveys with the mechanisms listed in the map.

**Objective:**

To inform existing data collection efforts and advance future research on the risk and protective factors relevant to adolescent suicide.

**Methods:**

We examined data from U.S. based surveillance systems and nationally-representative surveys that included (1) observations for an adolescent population and (2) questions or indicators in the data that identified suicidal ideation or suicide attempt. Using thematic analysis, we evaluated the codebooks and data dictionaries for each source to match questions or indicators to suicide-related risk and protective factors identified through a recently published suicide systems map. We used descriptive analysis to summarize where data were available or missing and categorized data gaps by social-ecological level.

**Results:**

Approximately 1-of-5 of the suicide-related risk and protective factors identified in the systems map had no supporting data, in any of the considered data sources. All sources cover less than half the factors, except the Adolescent Brain Cognitive Development Study (ABCD), which covers nearly 70% of factors.

**Conclusions:**

Examining gaps in suicide research can help focus future data collection efforts in suicide prevention. Our analysis precisely identified where data is missing and also revealed that missing data affects some aspects of suicide research (e.g., distal factors at the community and societal level) more than others (e.g., proximal factors about individual characteristics). In sum, our analysis highlights limitations in current suicide-related data availability and provides new opportunities to identify and expand current data collection efforts.

**Supplementary Information:**

The online version contains supplementary material available at 10.1186/s12889-023-15320-8.

## Background

Suicide is currently the second leading cause of death among adolescents ages 10–14, and third leading cause of death among adolescents ages 15–19 in the United States (U.S). Between 2009 – 2019, 26,194 deaths among adolescents aged 10–19 were suicides, constituting 28.3% of all deaths among those aged 10–14 and 25.2% of all deaths among those aged 15–19 [1]. Between 2009–2019, suicide rates among adolescents aged 10–19 years increased by 69.2% [1]. The strongest predictors of eventual suicide fatality are suicidal ideation or previous suicide attempt [2]. In 2018, an estimated 461,980 suicide ideation or attempt related emergency department visits and in-patient hospitalizations occurred among adolescents ages 10–19 [3]. The most recent data from the Centers for Disease Control and Prevention’s (CDC) Youth Risk Behavior Surveillance Survey show that 18.8% of high school students reported having seriously considered attempting suicide and 15.7% reported having made a suicide plan [4]. Data from the 2020 National Survey on Drug Use and Health show that nearly 3 million adolescents aged 12–17 (12.0%) had serious thoughts of suicide in the past year, 1.3 million (5.3%) made a suicide plan in the past year, and 629,000 (2.5%) made a nonfatal suicide attempt in the past year [5].

To address the public health challenge of steadily increasing suicide rates among adolescents [1–4], it is essential to understand what drives the increase in suicide ideation and implement interventions to reduce ideation and prevent attempts. Suicide, as with other forms of violence, has no single cause. Studies have identified multiple factors that contribute to adolescent suicide across all levels of the social-ecological model [6] (i.e., individual, relationship, community, societal), including mental health and substance use disorders, adverse childhood experiences (ACEs), social isolation, bullying and cyberbullying, gender and sexual minority status, availability of lethal means, neighborhood violence, and knowing someone who died by suicide [7–12]. The importance of identifying factors across all levels is that it guides policy makers and suicide prevention efforts towards the types of interventions that can address suicide risk at each level and helps researchers focus their data collection efforts towards specific areas at each level.

Recent articles took a key step towards achieving a comprehensive understanding of the many factors and causal pathways involved in suicide [13, 14]. These articles structured 361 suicide-related factors into a systems map, composed of 946 pairwise interactions. This map provides a qualitative, conceptual model of suicide in which we can trace potential causes and consequences. To further support evidence-based policies on suicide prevention, it is necessary to move into a quantitative stage that characterizes the prevalence of these factors and the strength of their interactions across populations and places. However, existing data on adolescent suicide risk and protective factors are fragmented across limited population-based surveys and surveillance systems, corresponding to the multifactorial nature of suicide. A comprehensive map is thus an opportunity to assess the availability of data across these sources. To date, there has not yet been a comprehensive assessment about the aspects of suicide for which we have data (across sources) and, most importantly, the aspects for which data is lacking. Consequently, this paper presents the first application of a systems map of suicide and ACEs in adolescents to identify data gaps and guide future data collection.

The remainder of this paper is structured as follows. Our methods explain how we identified four population-based surveys and one state-based surveillance system that collect information on adolescent suicidal behaviors and compared data from these sources with information from a previously released map [13, 14] available at https://osf.io/7nxp4/. Our supplementary materials provide detailed characteristics for these data sources (Table S1) and exemplify the process of matching their content with the map (Table S2). Our results summarize what is included and missing given the current data, both quantitatively (e.g., percentage of factors available in at least one survey) and qualitatively (e.g., which facets of suicide are missing the most). This analysis is performed both at the level of individual factors (e.g., availability of data on ACEs, social isolation) and connections between factors (e.g., availability of data on two connected factors within one survey), as an understanding of both levels is important to guide policy makers. The analysis also accounts for the social-ecological framework in distinguishing the availability of data at different levels (i.e., individual, relationship, community, societal) [6]. Detailed results for each of the 361 factors are also provided in Supplementary Table S3. Finally, we examine the implications of these results both from a systems science viewpoint and for public health.

## Methods

**Fig. 1:**
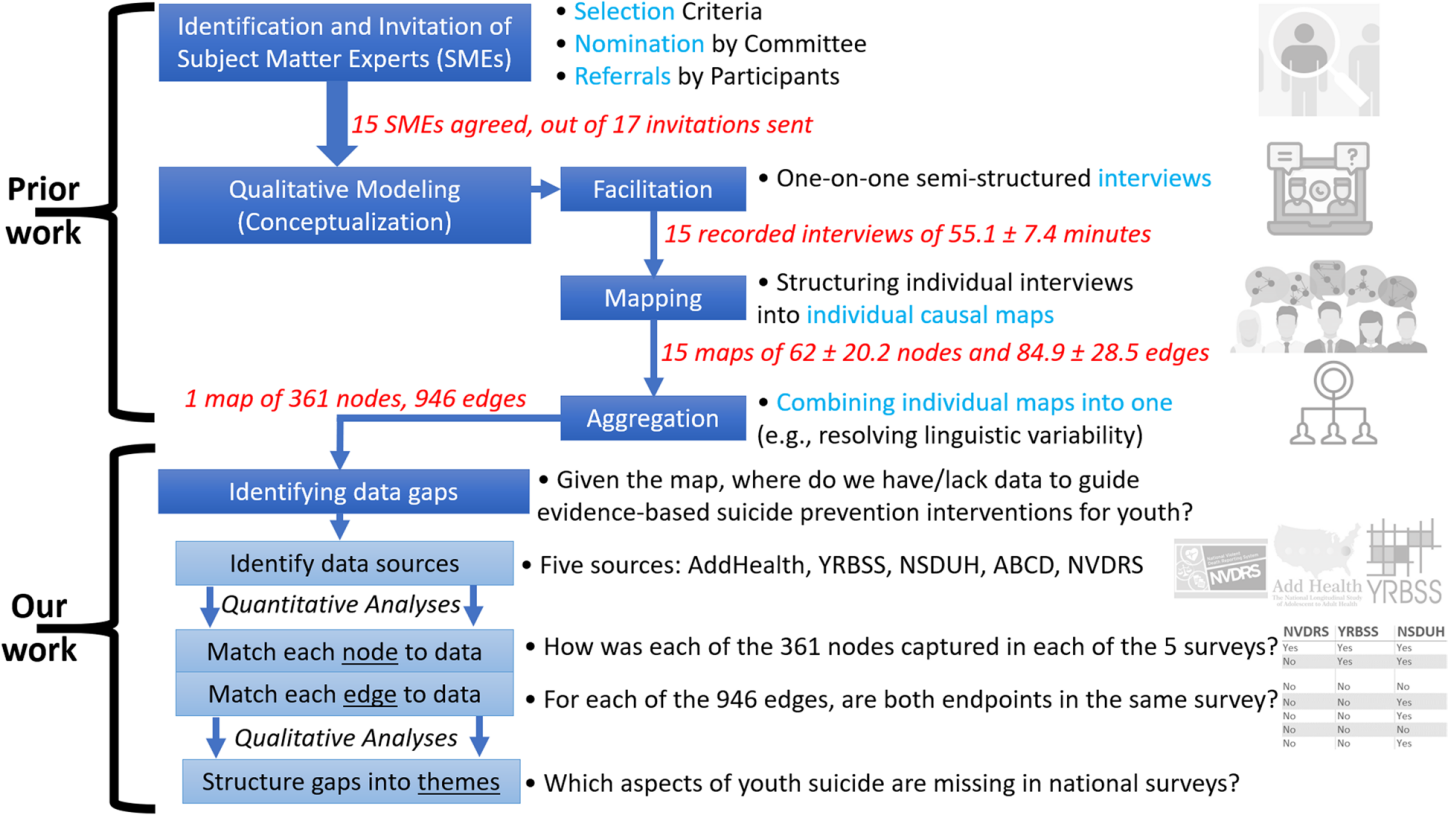
Overview of the methods. The upper portion was conducted in an open-access study [13], resulting in an open-access map. The bottom portion summarizes the methods of the present manuscript, starting with information from the map

A recent study published a systems map of adolescent suicide [13, 14], which was obtained by diverse facilitators who synthesized the knowledge from a diverse set of 15 subject-matter experts (SMEs). Each node in this map represents a suicide-related factor, such as devaluation of one’s identity, hopelessness, provision of care services, self-esteem, struggles in relationships, or trauma history. Each edge in the map states that one factor contributes to another, such as the impact of ACEs in the parents onto ACEs of their children, or the notion that capacity for suicide can be a precursor to suicide ideation. Our study uses this map as a starting point to determine whether its nodes and edges are covered by current data sources on adolescent suicidal behaviors (Fig. 1).

We started by identifying active, ongoing U.S. based surveillance systems or survey data sources that collect information on an adolescent population with any ages between 10–19. We examined each data source for two components often included in models of suicide: ideation and attempt [15–17]. To be included, a data source must contain a question or indicator that identifies if the adolescent expressed either present or past suicidal ideation or past suicide attempt. This includes survey data sources where such questions are directly asked to a participant, or surveillance data containing interviews (e.g., with family members) regarding circumstances preceding death by suicide. After identification of an ideation or attempt question or indicator, we then examined each data source to determine if observations in the data were drawn from a nationally representative sample of adolescents (representative across race and ethnicity, sex, and age) and if the surveillance data on adolescent suicide deaths included indicators of prior suicide ideation or prior attempts as preceding circumstances to the death. A full table of data sources considered and our selection process are provided in Supplementary Table S4. Given the selection criteria we identified five data sources for inclusion in the analysis. Characteristics of the data sources are detailed in Supplementary Table S1. The National Longitudinal Study of Adolescent to Adult Health (*AddHealth*) is a nationally representative longitudinal study of adolescents who were in 7th—12th grade during the 1994–95 school year, and have been followed for five waves to date, most recently in 2016–18. Given the retrospective nature of AddHealth, individuals who would now be in their 30’s and 40’s are asked to think back about their adolescence, hence the study achieves a representative sample for adolescents but would not qualify as an on-going adolescent health study. AddHealth includes questions across each wave related to the adolescent’s family and social environment, behavioral and cognitive health, and suicidal thoughts and behaviors [18]. The Youth Risk Behavior Surveillance System (*YRBSS*) is a school-based survey of representative samples of high school students (9th—12th grade), conducted biennially, and includes questions related to health-related risk behaviors, including suicidal thoughts and behaviors [19]. The National Survey on Drug Use and Health (*NSDUH*) is a nationally representative annual survey of youth and adults ages 12 and older, and includes questions related to drug use, behavioral and mental health, and suicidal thoughts and behaviors [20]. The Adolescent Brain Cognitive Development Study (*ABCD*) is a longitudinal study of brain development and child health and follows children from the ages of 9–10 through young adulthood. ABCD includes clinical and survey data related to social, emotional, and cognitive development, as well as a variety of health and environmental outcomes, including suicidal behaviors [21, 22]. Finally, the National Violent Death Reporting System (*NVDRS*) is a state-based surveillance system, collected annually, that captures all violent deaths, including suicides for all ages [23]. NVDRS uses three data sources: death certificates, medical examiner and coroner reports, and law enforcement reports. Trained abstractors write narratives describing any known characteristics and circumstances of the suicide decedent captured from law enforcement and medical examiner or coroner reports, including prior suicidal ideation and prior suicide attempts.

We examined the most recent years of data available for each source and qualitatively examined the content of codebooks and data dictionaries to identify any related questions or indicators that link quantitative data to each node identified through the systems map. For consistency and comparability of years examined, we started with 2016 for NSDUH due to its survey redesign, and examined up to the most current year of 2019. For YRBSS we included 2015, 2017, 2019 as it is collected biennially. For ABCD, we included the latest release available (version 3.0 [2020]), which includes data from the first release in 2019. For AddHealth we examined each wave as it is a longitudinal survey with the latest wave of data collected between 2016–2018. Finally, as NVDRS is the only active surveillance data set that captures suicide deaths, we examined data from each year available post the addition of the suicidal ideation and prior suicide attempt indicators in the NVDRS data set (years 2013–2018).

In order to appropriately capture relevant questions pertinent to the nodes, we searched on exact terms, associated terms (following edges to find drivers or consequences for each node), or synonyms related to each node when appropriate. For example, searches for factors related to housing stability included the synonyms, “homeless” and “eviction”. We used qualitative coding for each node to denote the presence (yes) or absence (no) of a question within each data source that could be used to link quantitative data to the node. Examples of this matching process are provided in Supplementary Table S2.

To exemplify this process within one source, consider the ABCD data, which is formed of over 300 data collection instruments, containing over 87,000 questions, variables, or fields. We used the National Institute of Mental Health Data Archive (NDA) online query tool available at nda.nih.gov, which allowed us to query all ABCD instruments using defined query categories. We thus performed manual queries on each relevant category, such as adverse events, depression, coping, loneliness, parenting, social responsiveness, or violence. We further focused on instruments that cover the full nationally-representative sample of the data, instead of instruments that were only administered on smaller samples of the ABCD population. Querying on these categories narrowed the ABCD data down to 18 data collection instruments, with a total of 2,061 questions, variables, or fields. We were then able to search on exact and associated terms within each instrument and denote the presence (yes) or absence (no) of a question within each data instrument that could be used to link quantitative data to the nodes identified through the systems map.

As a result of this matching process, we identify data gaps between the set of factors related to adolescent suicidal thoughts and behaviors and the existing data available to measure these factors. Building on this analysis, we also assess whether the two endpoint factors for each edge of the map are included in the same data source. Having data on two related factors within the same source is important to assess causes-and-effects, and thus estimate the potential impact of a suicide prevention policy. Finally, we take a thematic approach to summarize where data is available or missing with respect to the broader domains relevant in suicide research.

## Results

Our analysis for the presence or absence of a suicide-related factor across data sources is summarized in Table 1, while the detailed findings at the level of each factor (represented by nodes on the map) are provided in Supplementary Table S3. Similarly, our analysis on data for edges is summarized in Table 2, with scripts as a Python Jupyter Notebook available in Supplementary Material S4.

**Table 1:** Percentage of suicide-related factors available for a specific data source, no source, or in multiple sources

Data Source and Coverage	Percentage of suicide-related factors available (*n* = 364)
AddHealth	46.70% (*n* = 170)
NSDUH	38.46% (*n* = 140)
NVDRS	23.90% (*n* = 87)
YRBSS	15.38% (*n* = 56)
ABCD	69.23% (*n* = 252)
Covered by no data source	20.60% (*n* = 75)
In only one data source	22.80% (*n* = 83)
In ≥ 1 data source	79.40% (*n* = 289)
In ≥ 2 data sources	56.59% (*n* = 206)
In ≥ 3 data sources	35.44% (*n* = 129)
In ≥ 4 data sources	16.21% (*n* = 59)
All five data sources	6.04% (*n* = 22)

Categories are not mutually exclusive, hence totals exceed 100%

*AddHealth* National Longitudinal Study of Adolescent to Adult Health, *NSDUH* National Survey on Drug Use and Health, *NVDRS* National Violent Death Reporting System, *YRBSS* Youth Risk Behavior Surveillance System, *ABCD* Adolescent Brain Cognitive Development ^SM^ (ABCD) Study

**Table 2:** Availability of data at the level of causal mechanisms, i.e., edges that track the impact of one factor onto another

Coverage	Number of edges covered (*n* = 946)	% of edges covered
Within any one data source	755	79.81
Within AddHealth only	387	40.91
Within NSDUH only	272	28.75
Within NVDRS only	174	18.39
Within YRBSS only	85	8.98
Within ABCD only	645	68.18
Only covered by using different data sources (i.e., each endpoint of the edge must come from a different source)	17	1.79
Only one endpoint of the edge has data, across any data source	150	15.86
No data on either endpoint of the edge	22	2.32

Categories are not mutually exclusive, hence totals exceed 100%

*AddHealth* National Longitudinal Study of Adolescent to Adult Health, *NSDUH* National Survey on Drug Use and Health, *NVDRS* National Violent Death Reporting System, *YRBSS* Youth Risk Behavior Surveillance System, *ABCD* Adolescent Brain Cognitive Development ^SM^ (ABCD) Study

From Table 1, we note that approximately 1 of every 5 (20.60%) suicide-related factors has no supporting data, in any of the surveys. Most surveys cover less than half of the factors, with the exception of ABCD which covers almost 70%. ABCD also plays a major role when using multiple data sources, as it often provides data on aspects that are missing in all other surveys. Similarly, the large coverage of ABCD allows it to contain almost two thirds of the edges, making it a key source to study suicide-related factors. The second most comprehensive source, AddHealth, covers 41% of the edges, while other sources contain less than a third. We observe that it is difficult to study 1 of every 5 mechanisms as there is no single data source that tracks both aspects of the causal mechanism.

The availability of data is not necessarily uniform across suicide-related factors since the design of national surveys did not include questions uniformly across surveys. Consequently, we expect some aspects to receive more coverage than others, that is, data availability and gaps are likely to happen in clusters. Table 3 sheds light on this phenomenon by categorizing factors of the systems map alongside the social-ecological framework and reporting data availability for each level of the framework. Social-ecological levels include the individual (e.g., Ability to express oneself, Visit to ER, Delinquency), relationships (e.g., Bullying, ACEs of siblings, Domestic violence), community (e.g., Community exposure to suicide, Community promotion of mental health), and societal level (e.g., Cultural norms that do not see ACEs as a problem, Economic policies for ACEs). The map itself is focused on the individual, with fewer factors as we move towards a more societal level. Even when taking this focus into consideration, we note that the percentage of data available strictly decreases as we move away from the individual, down to less than half of factors covered at the societal level.

**Table 3:** Prevalence of variables by social-ecological level

*Number of variables*	*Sociol-ecological level*			
	Individual	Relationship	Community	Societal
*In adolescent suicide systems map *[13]	152	145	99	55
*In at least one data source*	132	121	73	26
*Coverage of the map given the data sources (%)*	86.84	83.45	73.74	47.27

## Discussion

### Key findings

Over approximately a decade, there has been a 44% increase in suicide planning [24] and a 57% increase in suicide rates among adolescents and young adults ages 10–24 [25]. Several of these factors have recently been on the forefront, for example due to the youth mental health crisis [26]. Adolescent suicide is a complex phenomenon, shaped by a large number of interacting causes across multiple levels of the social ecology [6, 27]. In addition, suicidal thoughts or attempts also have consequences that can fuel some of these causes, thus creating feedback loops. To identify and evaluate interventions that address suicide risk, it is thus essential to take a systems approach [28]. A first step is the development of a systems map, which articulates a comprehensive list of suicide-related factors and their interactions. Such a map complements established theories of suicide [29, 30] by providing a fine-grained view of a complex system. Building on the recent release of a systems map of suicide [13, 14], our study focuses on the second step to support evidence-based policymaking and suicide prevention efforts: using the map to assess where data is available and, most importantly to focus future data collection efforts where data is currently lacking. Our study thus applied a systems science approach to identify gaps in data availability on adolescent suicidal ideation and suicide attempt. As suicide is currently the second and third leading cause of death among adolescents in the United States [1], this study provides a first look at where data is needed to examine multiple risk factors associated with increased rates of adolescent suicidal behaviors.

This effort echoes previous undertakings at the intersection of public health and systems science. For instance, in the field of obesity research, the initial release of a map [31] contributed to reframing obesity as a complex problem [32] and led to several follow-up analytical studies [33, 34]. One such analysis in obesity research focused on the availability of data [35], but it was limited to nodes of the map and was driven by members reporting the possibility of applying new data sources (e.g., supermarket loyalty cards, apps and wearables). Our study, which is the first to perform this assessment for suicide, extends the methodologies of prior assessments in fields such as obesity in several ways. First, we established a direct match between factors in the map and factors that are currently collected in national surveys; these direct linkages can readily help analysts and are provided as supplementary materials. Second, we did not only examine data availability on factors in isolation; rather, we continued a systems approach by also looking for quantitative evidence regarding relations between factors, which is essential to examine suicide-related factors and thus crucial for policymaking. Finally, we took a thematic approach to nuance the availability (or lack) of data based on the social-ecological framework, which ranges from individual factors to societal considerations. The variables present in all surveys are primarily demographic (e.g., race and ethnicity, child age, sexual minority) and those most often shared by surveys are common individual-level mechanisms such as access to lethal means, capacity for suicide, high-level child risk factors, clarity of planning, disruptive behaviors, exposure to violence, or interpersonal problems and stressors. In contrast, many essential variables do not appear in any of the data sources, with a disproportionate lack of data for community-level factors (e.g., exposure to suicide, promotion of mental health, resources for health), and societal-level factors (e.g., culture of secrecy, valuing a sense of independence as self-reliance for health needs). Quantitatively, there is a clear gradient between the availability of data and the socio-ecological framework, whereby data coverage strictly decreases as we move away from the individual and into more societal-level factors. Once we move beyond a child’s immediate characteristics, we quickly lack data on the family (e.g. parental involvement in care), community, and social environment in which trauma and suicide happen. The lack of data is compounded when we take a systems approach to track relations. For instance, three data sources recorded an identity conflict, but only one examined whether the individual’s identity was socially accepted or approved in the community, which can be one of the roots for an internal conflict. This over-emphasis on the individual limits our ability to understand how complex situations are shaped by the environment, despite the fact that our policies will have to act through this environment before being able to reach individuals.

The situation may gradually be changing with the emergence of newer data sources that provide significantly more coverage. For example, the ABCD consortium was funded in 2015, and the eponymous study covers almost 50% more factors or relations than the next most comprehensive data source (AddHealth). For instance, ABCD covers factors that are captured in no other data source, such as hospitalization from abuse, suicide contagion, or an increased willingness to apply pain to oneself. Assessments such as presented here are thus critical to guide the development of future data collection efforts and ensure that data coverage continues to increase. As such, our systems process can be replicated as new years of data are collected to continue to monitor trends in data availability as levels of risk in communities and societies change.

Collection of nationally-representative population level data on suicide is inherently challenging since population health surveys are significant data collection efforts and not every question may be asked in a general instrument. The fact that a survey such as AddHealth covers almost half of suicide-related factors is noteworthy, given its wide focus. Although suicide research would benefit if data sources covered all risks and protective factors, the demands placed upon nationally representative surveys may preclude them from covering 100% of constructs. By showing that some categories of constructs are more neglected than others, our study thus contributes to assessing areas where data is most needed in order to prioritize efforts.

### Data limitations: from the existence of data to analyses and generalizability

Our assessment is an important first step to identify whether data *exists* regarding suicide-related factors and their relationships. Non-existing data would prevent us from conducting analyses at the population level and could help determine priorities for future data collection efforts. However, the existence of data does not entail that all analyses can be supported and that findings can automatically be generalized. This subsection thus nuances our findings by emphasizing some of the limitations within existing datasets, which can also contribute to guiding future data collection efforts.

In the data sources, population weights are used to provide nationally representative samples. For example, this allows findings derived from these sources to be generalized in terms of race and ethnicity. However, as highlighted by Cha and colleagues in their review of diversity in suicide research [36], generalization is better supported on demographic factors such as age, sex, race and ethnicity, than on aspects such as gender identity and sexual orientation [37, 38]. For instance, ABCD does not ask about sexual preference given the young ages of the participants. A similar problem arises with respect to socioeconomic status, as research on family economic security and suicide may be dated, have conflicting findings, or report the absence of a causal effect. For example, the work of Braudt et al*.* on the 1998–2015 National Health Interview Survey-Linked Mortality Files (NHIS-LMFs) found no relationship between parental socio-economic status and death by suicide in children and youth [39]. In addition, ABCD being a ten-year longitudinal study launched in 2018, it currently only covers children hence generalization to adolescents would only be possible when later years of data collection are completed.

Furthermore, even if a construct is covered in a sample that generalizes with respect to the features of interest to a researcher, this construct may not be measured in a way that supports a desired analytical approach. For example, AddHealth and YRBSS both cover the links between family economic security and suicide, but they do not measure them in the same manner.

Researchers interested in analyzing suicide-related factors but who do not require nationally representative data over multiple criteria may access additional datasets (e.g., as shown in Supplementary Table S4) as well as other studies such as qualitative and ethnographic research. These sources also contribute to informing theories of suicide and scientific knowledge about suicide [40, 41]. It is also important to note that datasets that are not nationally representative of the entire U.S. population have significant values for suicide research. For example, the National Latino and Asian American Study (NLAAS) is critical to understand the diverse and intersectional experiences of mental health in these populations [42]. To produce a robust understanding of suicide in the U.S. across all youth, future studies may consider datasets that are representative of specific populations. Intersectional analyses involving such sources [43–46] can contribute to preserving the variation that may be lost when smaller demographic groups are diluted within a large population, as discussed in the context of small minoritized subpopulations such as Native Americans [47].

## Conclusions

Our assessment highlights limitations in researchers’ ability to link risk and protective factors with suicidal ideation and attempt. No available data source includes a comprehensive inventory of all risk and protective factors for adolescent suicide. Still, a growing body of research suggests that systems-level interventions, such as policies that promote household economic stability (e.g., minimum wage, tax credits) are likely to impact both adverse childhood experiences, child mortality, and suicide [35]. Innovative data that links risk and protective factors, mental health, and suicide could enhance our ability to understand other policies or system changes that may create safe, stable, nurturing environments for children and lead to long term reductions in suicide [48].

Recently NIH and the National Center for Health Statistics (NCHS) have taken steps to address some of the challenges by creating a number of surveillance and monitoring systems that can be linked directly with mortality data. Through the Adolescent Brain and Cognitive Development Study, NIH is greatly increasing the ability to link contextual data with adolescent health outcomes, including biomarkers for brain and physical development. These new data sources offer new opportunities to identify interventions that can potentially prevent ACEs, promote resilience, and prevent suicide across the lifespan.

## Supplementary Information

**Additional file 1:** **Table S1.** Characteristics of the data sources.

**Additional file 2: Table S2.** Examples of matching sources.

**Additional file 3:** **Table S3.** Availability of data for each of the concept nodes.

**Additional file 4.** Scripts for link-level analysis.

**Additional file 5:** **Table S4.** Suicide datasets inventory.

## Data Availability

All data generated during this study are included in the supplementary information files. Specifically, these supplementary online materials include the detailed results at the node level as well as Python scripts in a Jupyter notebook to obtain results at the level of edges.

## Abbreviations

ACEs: Adverse Childhood Experiences
SMEs: Subject Matter Experts
