# Supplementary figures and images for: A systems science approach to identifying data gaps in national data sources on adolescent suicidal ideation and suicide attempt in the United States
Source: BMC Public Health. 2023 Apr 1;23:627. doi: 10.1186/s12889-023-15320-8 (PMC10067278; doi:10.1186/s12889-023-15320-8)

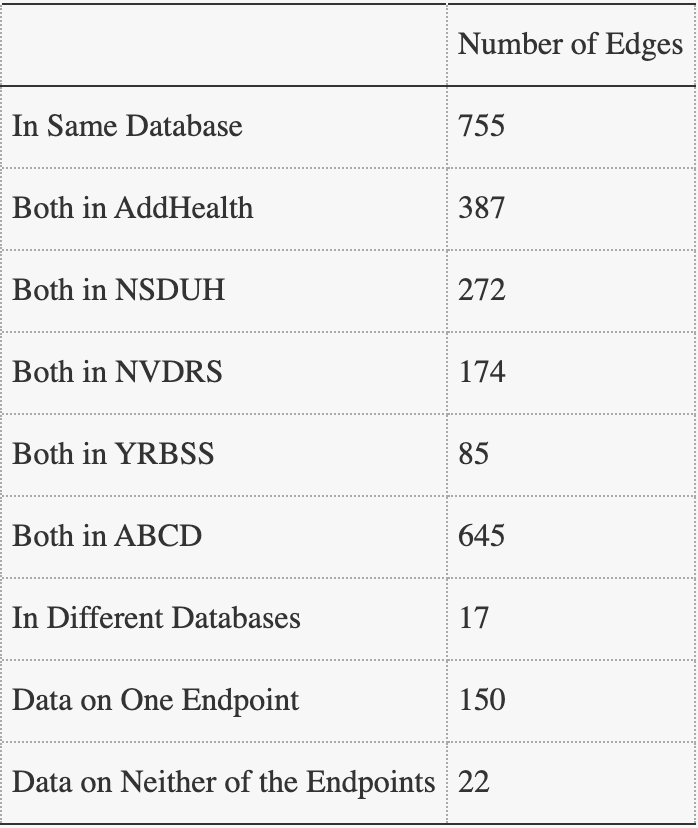

Supplement: Supplementary file 4 — Additional file 4. Scripts for link-level analysis. [file 12889_2023_15320_MOESM4_ESM.zip › Edge Classification/output.png]
